# Wetting properties of blood lipid fractions on different titanium surfaces

**DOI:** 10.1186/s40729-020-00213-x

**Published:** 2020-05-13

**Authors:** Revan Birke Koca, Onur Güven, Mehmet Sabri Çelik, Erhan Fıratlı

**Affiliations:** 1 Department of Periodontology, Faculty of Dentistry, University of Kyrenia, 99320 Kyrenia, Cyprus; 2 Department of Mining Engineering, Faculty of Engineering, Adana Alparslan Türkeş Science and Technology University, Adana, Turkey; 3 Department of Mineral Processing Engineering, Faculty of Mines, İstanbul Technical University, İstanbul, Turkey; 4 Harran University Rectorate, Şanlıurfa, Turkey; 5 Department of Periodontology, Faculty of Dentistry, Istanbul University, İstanbul, Turkey

**Keywords:** Animal model, Cholesterol, Contact angle, Hyperlipidemia, Implant surface, Statin, Wettability

## Abstract

**Background:**

Blood is the first tissue contacting the implant surface and starting the biological interactions to enhance osseointegration and stimulate bone formation with the progenitor cytokines, chemokines, and growth factors. The coagulation cascade initiates the first step of osseointegration between implant and neighboring tissues. The wound healing may be inadequate unless the blood wets the implant surface properly. Wettability is one of the most important features of the implant surface while lipid level constitutes a milestone that may change the energy of blood, which determines its distribution on implant material. Thus, the aim of this study was to evaluate the effect of lipid component of blood as cholesterol and its treatment on their wetting behavior of titanium surfaces.

**Methods:**

Five surface groups were formed including grade 4 titanium-machined, grade 4 titanium-SLA, grade 4 titanium-SLActive, Roxolid-SLA, and Roxolid-SLActive. In healthy, hyperlipidemic, and treatment situations, blood was taken from eight rabbits and dropped to the disc surfaces. Contact angles were measured between the blood samples and disc surfaces.

**Results:**

A significant difference was found between both machined and SLActive surfaces, SLA and SLActive surfaces in the hyperlipidemic period, and only Roxolid-SLA and SLActive surfaces during the treatment period. When evaluated according to time, only grade 4-machined and Grade 4-SLA surfaces showed a significant difference.

**Conclusions:**

Our findings indicated that each period has its own characteristics and showed the importance of cholesterol in blood structure on applicability of implant surfaces.

## Background

Titanium is a light, mechanically, and chemically resistant material, which is durable enough to handle large loads and does not break despite the bending. Due to their near-perfect biocompatibility, titanium and its alloys are still the first choice materials for dental implants [1]. Pure titanium can be found in four forms as grade 1-4 and contains between 0.18 and 0.4% oxygen [2]. On the other hand, a diversity of types can be found for their alloys (titanium grade 5, Ti-6Al-4 V, Roxolid [3], etc.).

Besides their other chemical and mechanical properties, the surface characteristics of the implants such as roughness trigger the most important role during that the rate of osseointegration may be in line with the roughness degree of implant [4, 5]. Various physical, mechanical, chemical, or biochemical modifications, could be applied together or separately to enhance the surface topography and roughness for amplifying the tissue response [6–8]. In this manner, while machined ones present smoother surfaces [9, 10], the sandblasted and acid-etched ones present rougher surfaces with their corresponding features in terms of physical and chemical properties related to the bone-implant interfaces [11, 12].

Thus, blood is the first tissue that is associated to the implant surfaces and starts the biological interactions to enhance osseointegration with the progenitor cytokines, chemokines, and growth factors to stimulate a new bone formation [13]. Therefore, the wetting behavior of blood becomes very important that if the blood does not wet the implant surface properly, the wound healing may be inadequate. On the other hand, coagulation of blood (clotting) is the first step of osseointegration between implant and neighboring tissues, which are significantly affected by their wetting behavior [14]. In this manner, contact angle (CA) is the most suitable method for measuring the compatibility between blood and implant surface interfaces in terms of wetting behavior of blood and wettability of implant surfaces [15]. Although many studies have been adapted to show the effects of low or high density lipoproteins of bloods on viscosity and accordingly their effects on wetting behavior [14, 16], only a few of them included the role of hyperlipidemia which is known to adversely affect osseointegration by suppressing osteoblast differentiation [7, 17–19]. On the other hand, the treatment of hyperlipidemia is another concept that the most effective way is the usage of statins, which are HMG-CoA reductase inhibitors [20, 21] and known to decrease wetting behavior of blood in accordance with their effects on viscosity. Thus, in this study, the variations on wetting behavior of blood samples before and after treatment of hyperlipidemia with statins were investigated by conducting contact angle measurement on commercially available implant surfaces.

## Materials and methods

In this study, the rabbit feeding with specific diets was carried out in Istanbul University Aziz Sancar Experimental Medicine Research Institute Division of Laboratory Animals Science, the contact angle measurements were performed at the Surface Chemistry Laboratory at Department of Mineral Processing Engineering, Istanbul Technical University. The study was conducted by the Istanbul University Animal Experiments Ethics Committee with the approval of 35980450-050.01.04.

### Experimental animals

Eight male New Zealand rabbits in the range of 3.5-4.5 kg body weight and 8–10 months aged were obtained from İstanbul University Aziz Sancar Experimental Medicine Research Institute, Laboratory Animal Science Department. They were accommodated in steel cages in an ambient temperature of 25–30 °C and 45–50% relative humidity with 12 h light and dark cycles per day. The rabbits were also numbered in order to evaluate the responses of different periods individually.

### Animal groupings and experimental methods

In experimental studies, single groups of experimental animals were evaluated in an order of three periods as healthy, hyperlipidemic, and treatment periods.

#### Healthy period

In this period, no intervention was made for 2 weeks from the beginning of the study for the adaptation of rabbits.

#### Hyperlipidemic period

In this period, animals were made “hyperlipidemic” by a cholesterol-rich diet for 5 weeks.

#### Treatment period

Following the hyperlipidemic period, the animals were treated with statin for 5 weeks.

#### Disc surfaces

As mentioned in the “Introduction” section, five commercially available and clinically applied discs with Ti or Ti-zirconium surfaces were used. In experimental studies, in order to make a reliable evaluation, each disc type was also coded with a group symbol such as grade 4 machined surface (group A), hydrophobic grade 4 (sand-blasted, roughened with acid, SLA®) (group B), hydrophilic grade 4, (sand-blasted, roughened with acid and chemically modified with N_2_ gas then preserved in isotonic NaCl solution, SLActive® (group C), hydrophobic Ti-Zr alloy (with 15% Zr, Roxolid-SLA®) (group D), and hydrophilic Ti-Zr alloy (with 15% Zr, Roxolid-SLActive®) (group E).

### Experimental procedure

**Fig. 1:**
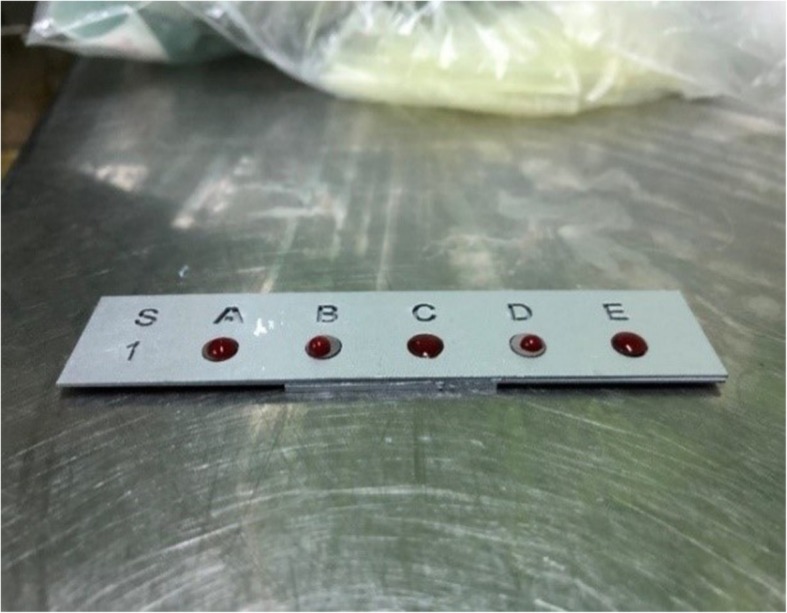
Blood samples before clotting

Rabbits were fed with a normal diet for 2 weeks ad libitum. At the end of 2 weeks, each animal was weighed and their weight was recorded. Blood samples were collected using blood collection tubes with 9-ml empty and 5-ml separator gel containing tubes equipped with 23 G needles from the marginal vein in the ears of each rabbit and immediately transferred to the lab. The automatic pipette was placed in a custom-made device, and blood samples were equally (30 μl) dropped on to the surfaces of sterile discs (5 mm diameter, 1 mm height) with different surface properties (Fig. 1).

**Fig. 2:**
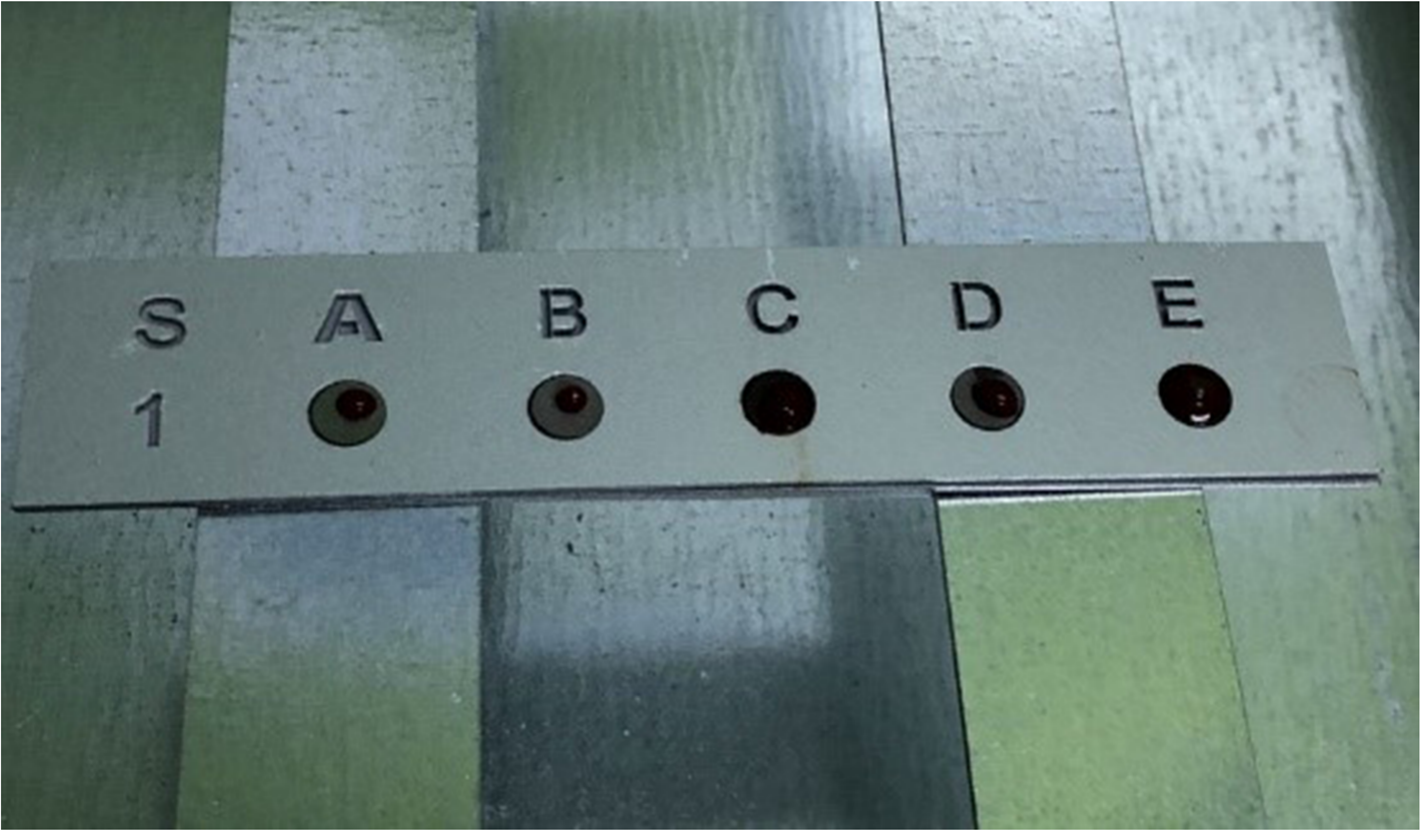
Blood samples after clotting

The tubes containing the separator gel were centrifuged at 4500 rpm for 5 min. The colorless serum in the upper part of the tubes was extracted with insulin injectors and transferred to 4-ml Eppendorf tubes. An empty blood collection tube containing a separator gel and an Eppendorf tube for each animal were used and numbered according to the code of animals. As mentioned in the “Introduction” section, in order to investigate the effects of different components on osseointegration with wetting behavior of blood samples, the contact angle measurements were utilized following their clotting. Considering that the discs with clotted blood samples (Fig. 2) were transferred to the Surface Chemistry Laboratory in the Department of Mineral Processing Engineering at Istanbul Technical University for contact angle measurements. The contact angle was measured between the clot and each surface with a goniometer (OCA15EC model SCA 20 software, DataPhysics Instruments GmbH, Filderstadt, Germany). The values were recorded in degrees. Since the device measured the contact angles of the drops separately from the right and left positions, the arithmetic mean of the values was recorded.

Meanwhile, the serum fluids in the Eppendorf tubes were transferred to the laboratories of Istanbul University Veterinary Faculty. Total cholesterol and triglyceride levels were measured (total cholesterol and triglyceride kits - MVM Medical, İstanbul, Turkey). All values obtained were recorded in mg/dL.

After 2 weeks of adaptation time, 5-weeks hyperlipidemic period started. For the hyperlipidemic diet, 2% hypercholesterolemic pellet feed rich in fat (MBD Feed Trade©, Kocaeli, Turkey) was prepared with 92.5% of powder cholesterol (Sigma-Aldrich®, Taufkirchen, Germany). As mentioned in the previous section, the animals followed a hyperlipidemic diet for 5 weeks while no animals were intervened.

Blood tubes were centrifuged under the same conditions, and serum fluid was transferred to numbered Eppendorf tubes. Eppendorf tubes were taken to the center, and blood cholesterol and triglyceride levels were measured. Following the hyperlipidemic period, rabbits were transferred to the normal diet, for this period, 3.7 mg/kg atorvastatin powder per day was added to their diet for 5 weeks. The rabbits were weighed day by day, and the statin dose was calculated for each animal according to their weight on a precision scale (KERN & Sohn GmbH, Balingen, Germany). The drug was suspended and applied with gavage. In this way, treatment was initiated to bring the total cholesterol and triglyceride levels to the threshold value. At the end of this procedure, the same procedure was re-administered, and the values of blood-surface contact angle and blood total cholesterol and triglyceride levels were recorded.

### Statistical analysis

Data were analyzed on the computer using SPSS 25.0 (Statistical Packages of Social Sciences). The conformity of the data to the normal distribution was evaluated with the Kolmogorov-Smirnov test, and descriptive statistics were shown as mean ± standard deviation, median, minimum and maximum values for continuous variables. While the homogeneity of the variance between the groups was evaluated by the Levene test, the Welch ANOVA test was used to compare the non-homogeneous measurements of the variance between the groups, and the Dunnett’s T3 test was used as a post hoc test for statistically significant measurements. The Kruskal-Wallis test was used to compare the measurements that did not comply with the normal distribution among the groups, and the Mann-Whitney *U* test was used as the post hoc test of the statistically significant measurements. The results were interpreted with Bonferroni correction. Variations in the variables that did not conform to normal distribution within the group were evaluated by Friedman test. The time-dependent change was statistically significant, and the post hoc test-dependent Wilcoxon test was used, and *p* values were interpreted by Bonferroni correction. In the case of *p* < 0.05, the difference was considered significant.

## Results

### Estimation of serum lipid levels

**Fig. 3:**
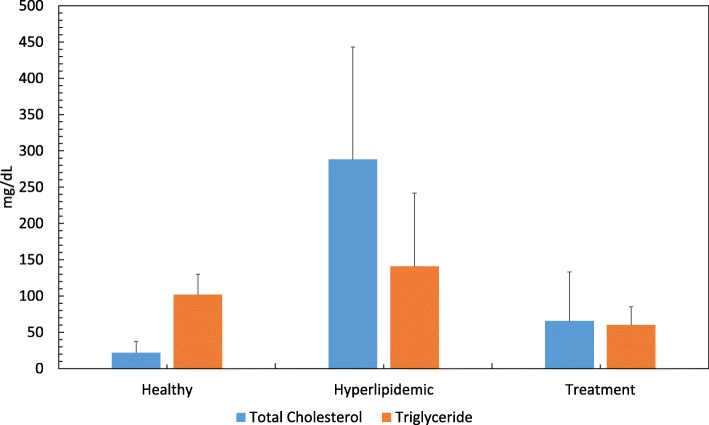
Serum total cholesterol and triglyceride levels by periods

Total cholesterol levels of experimental animals increased in the hyperlipidemic phase and decreased in the treatment phase significantly (*p* < 0.001). Although triglyceride levels are also proportional to total cholesterol values, the changes are not significant. Serum total cholesterol and triglyceride levels of rabbits appear in Table 1 and Fig. 3.

**Table 1:** Serum total cholesterol and triglyceride levels

		Mean ± Std Dev.
Total cholesterol, mg/dl	Healthy	21.83 ± 15.61
	Hyperlipidemic	288.33 ± 154.81
	Treatment	65.50 ± 67.56
Triglyceride, mg/dl	Healthy	101.91 ± 28.01
	Hyperlipidemic	140.66 ± 101.26
	Treatment	60.125 ± 25.03

### Contact angle measurements

**Fig. 4:**
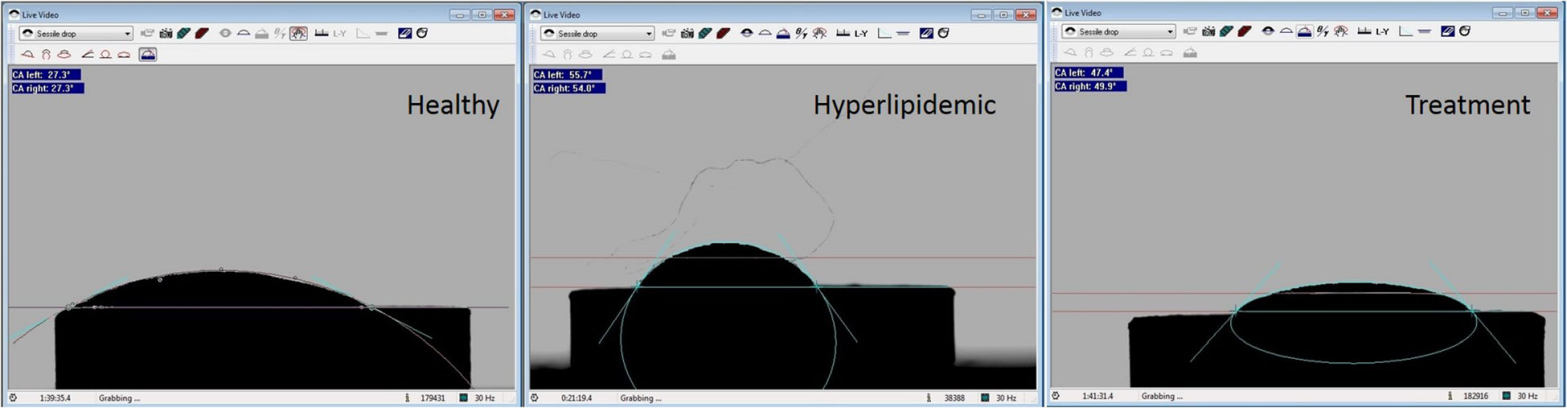
Contact angle images of the same sample in healthy, hyperlipidemic, and treatment periods

**Fig. 5:**
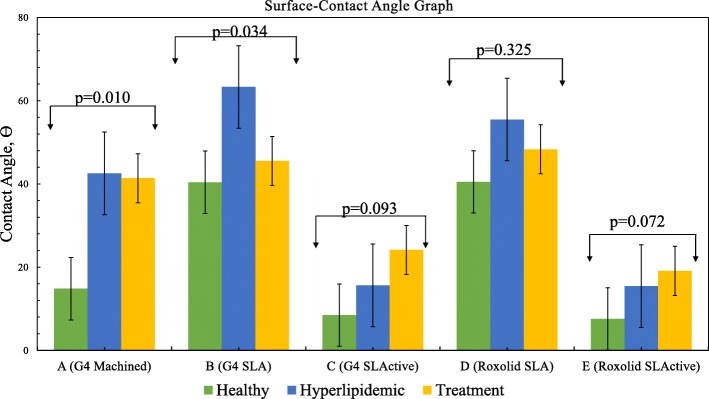
Contact angles at different titanium surfaces for different treatment periods

A representative contact angle image analysis in all periods of blood samples was shown in Fig. 4. In addition, graphical presentation of contact angle as a function of periods was shown in Fig. 5. Contact angles of groups in all periods are given in Table 2.

**Table 2:** Comparison contact angles formed on different discs in all periods (*p* < 0.05)

Group		Healthy	Hyperlipidemic	Treatment	*p* value
A	N	8	8	8	0.010*
	Mean	14.8	42.5	41.4	
B	N	8	8	8	0.034*
	Mean	40.4	63.3	45.5	
C	N	8	8	8	0.093
	Mean	8.5	15.6	24.1	
D	N	8	8	8	0.325
	Mean	40.5	55.5	48.3	
E	N	8	8	8	2
	Mean	7.6	15.4	19.1	

According to the results given in Table 2 and Fig. 4, in the healthy period, while the contact angle values were measured as 40.4° and 40.3° on hydrophobic B and D surfaces, the values remained as 14.8°, 8.5°, and 7.6° for hydrophilic A, C, and E surfaces. As mentioned in the “Introduction,” these results also indicated that in addition to their hydrophilic or hydrophobic features, the spreading of blood on implant surfaces might vary based on the surface characteristics like roughness or any kind of topographic variation by their production method. However, following the changes on blood structure by hyperlipidemic diet, higher contact angle values were obtained regardless of surface type. Therefore, in addition to morphological variations, if we consider the values of lipid fractions in blood samples (see Table 1), these findings could be ascribed to the increasing values of them (like total cholesterol and triglyceride) in blood structure which determines the energy and thus wetting behavior of blood. As a result, the spreading of blood on surfaces would be lower compared to its original form as expected.

In the treatment period, only the D group had a significantly higher contact angle than the C and E groups. The explanation for this phenomenon is that the Roxolid SLA surfaces represented by the D group have a higher contact angle than the SLActive surfaces for each period. As can be seen in Table 2, the change in blood lipid properties for Group D does not make a significant difference. In the hydrophilic groups (C and E), the contact angle with the treatment is increased, but this is not a statistically significant increase.

As mentioned in the “Introduction” section, if a statistical evaluation was carried out for each implant group in whole periods, the mean angle values shown in Fig. 5 could be used.

In the healthy period, the mean angle of group B was higher than group C statistically. The mean angle of group B was higher than group E. The mean angle of group D was higher than group C. The mean angle of group D was higher than group E.

In the hyperlipidemic period, the mean angle of group A was higher than group C statistically(^*^). The mean angle of group A was higher than group E. The mean angle of group B was higher than group C. The mean angle of group B was higher than group E. The mean angle of group D was higher than group C. The mean angle of group D was higher than group E. In the treatment period, the mean angle of group D was higher than group C statistically. The mean angle of group D was higher than group E.

**Fig. 6:**
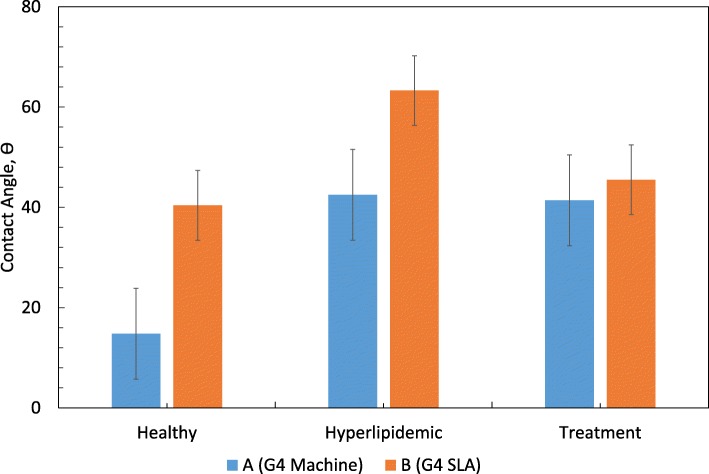
Evaluation of the groups according to the period and double comparisons of group A and B, *p* value by period

Only in A and B groups (Fig. 6), the change of contact angle was found to be statistically significant with respect to time. There is a significant difference between healthy and hyperlipidemia periods and between healthy and treatment periods in group A statistically. Although there was a significant difference in the change of contacts with the disc in group B, there was no statistically significant difference between the two comparisons.

Although there were higher angles in the other surface groups (C, D, and E) in the hyperlipidemic period than healthy and treatment, no significant difference was observed, indicating that the effect of hyperlipidemia or statin on wettability was not significant.

## Discussion

Rabbits are frequently used as experimental animals in hyperlipidemia and atherosclerosis studies since it is possible to create a fast and human-like hyperlipidemia model in rabbits by diet [20]. When other experimental animals are examined, mice form a different hyperlipidemic model from human in terms of deficiency of cholesteryl ester transport protein (CETP). This protein is needed for the formation of LDL and VLDL in humans. In the sera of dogs, rats, and pigs, no increase in CETP enzyme activity is observed, similar to mice [21]. Male rabbits were used in this study to prevent any hormonal difference from affecting lipid metabolism during the experiment period. The duration of the hyperlipidemic diet applied to experimental animals has been determined according to various studies [22]. In the same way, certain studies have been examined for dose calculation of statin given during treatment. According to one of these articles determined for the dose calculation of statin given to the experimental animals during the treatment period, the ratio of human/rabbit metabolism was specified as 0.07 [23]. According to another literature, the statin treatment dose in humans was determined to be 80 mg/day [24]. Because of that statin dose was calculated as 3.7 mg/kg/day by multiplying 0.07 in the study.

Dental implants are highly successful in healthy patients, but further innovations are needed to improve performance in compromised patients. This study is the first to evaluate the effect of hyperlipidemia and statin on the wetting behavior of blood samples and their compatibility with commercially available dental implant surfaces with different surface properties.

Measurements were made with clotted blood drops. These drops typically spread, and sometimes the peak may collapse slightly. This is due to the precipitation of thrombus formed during the coagulation of blood and the adhesion of plasma proteins to the surface [25]. Heparin, which can be used to prevent blood clotting, is avoided because it affects the contact angle and may mislead the study [26]. At the same time, heparin can dissolve the LDL (low density lipoproteins) in the blood which is dripped on the surface and may cause misleading results in lipid measurement [27]. The study was therefore carried out without the addition of any substrate to the blood tubes, and the measurements with blood provide more accurate findings compared to those of the measurements with distilled water.

On the other hand, the results also showed that the lowest contact angles were measured on the most hydrophilic surfaces “SLActive” as expected. The fact that it is made of grade 4, or Roxolid materials does not make a significant difference. There was a significant difference between the machined surface and grade 4 SLA groups by period. However, in the grade 4 SLA group, double periodic differences were not significant, and the difference was observed when evaluated as a whole. Accordingly, the effect of hyperlipidemia and statin on wettability was significant only in the machined surface group.

In literature, a study made on the contact angles between machined, SLA, and SLActive discs, and distilled water revealed the contact angle on the SLA surfaces is significantly higher than the machined and SLActive surfaces [28], and the lowest contact angle was found on SLActive surfaces, which were in line with ours. Another research on contact angle variations reported on SLA, SLActive, and Roxolid SLActive surfaces showed that while SLA surface was found hydrophobic, while SLActive and Roxolid SLActive were found hydrophilic [29]. These results also comply with our results.

The average contact angle values in the hyperlipidemic period were significantly higher than the values measured on the healthy and treated periods.

Contact angles measurements revealed that the average values in the hyperlipidemic period were significantly higher than the values measured on the healthy and treated periods. As mentioned in relevant sections, following the increase of lipid fractions like cholesterol in blood structure, higher contact angle values were obtained as expected. It is worth no note that despite the energy level being partially maintained (possibly created by the level of lipid fractions in blood) even after the treatment period, and it provided relatively higher contact angle values compared to its healthy state. According to the measurements made after healthy, hyperlipidemic, and treatment periods, the values measured in healthy condition could not reach the required value even after the drug treatment applied in proportion to the blood cholesterol level.

Considering that findings, this study provided a breakthrough in the literature to examine the effects of both hyperlipidemia and its treatment by statins on wetting behavior of blood samples on implant surfaces.

## Conclusion

In this study, the effects of hyperlipidemia and its treatment processes on the wettability of different implant surfaces were investigated by contact angle measurements. The results obtained that regardless of the physical properties of the surfaces, the surfaces become less wettable by clotted blood samples at elevated triglyceride and cholesterol levels in the hyperlipidemic diet for the first time.

In the literature, there is no study evaluating the effect of both hyperlipidemia and statin on wettability by using surfaces in this study. The original value of this study is that it is the first and only study using hyperlipidemic blood as wetting fluid between contact angle studies and it demonstrates quantitatively that the hyperlipidemic blood lipid level cannot be reduced to its healthy state even after treatment. In this way, the results of this study uniquely showed that even after the treatments with statin, the wetting behavior or energy level of the blood could not be reduced to that in the healthy state, which in turn determines the spreading of blood on implant surfaces.

## Data Availability

The datasets used and/or analyzed during the current study are available from the corresponding author upon reasonable request.

## Abbreviations

μl: Microliter
Al: Aluminum
CA: Contact angle
dl: Deciliter
G: Gouge, measurement of needle
HMG-CoA: 3-hydroxy-3-methylglutaryl-coenzyme A
kg: Kilogram
LDL: Low density lipoprotein
mg: Milligram
ml: Milliliter
mm: Millimeter
NaCl: Sodium chloride
Rox-SLA: Roxolid sandblasted acid-etched
Rox-SLActive: Roxolid modified sandblasted and acid-etched
Ti: Titanium
V: Vanadium
Zr: Zirconium
